# Biocompatible natural deep eutectic solvent-based extraction and cellulolytic enzyme-mediated transformation of *Pueraria mirifica* isoflavones: a sustainable approach for increasing health-bioactive constituents

**DOI:** 10.1186/s40643-021-00428-9

**Published:** 2021-08-17

**Authors:** Fonthip Makkliang, Boondaree Siriwarin, Gorawit Yusakul, Suppalak Phaisan, Attapon Sakdamas, Natthapon Chuphol, Waraporn Putalun, Seiichi Sakamoto

**Affiliations:** 1 School of Languages and General Education, Walailak University, Nakhon Si Thammarat, Thailand; 2 Faculty of Pharmaceutical Sciences, Huachiew Chalermprakiet University, Samut Prakan, Thailand; 3 School of Pharmacy, Walailak University, Nakhon Si Thammarat, 80160 Thailand; 4 Biomass and Oil Palm Center of Excellence, Walailak University, Nakhon Si Thammarat, Thailand; 5 Faculty of Pharmaceutical Sciences, Prince of Songkla University, Songkhla, Thailand; 6 Faculty of Pharmaceutical Sciences, Khon Kaen University, Khon Kaen, Thailand; 7 Graduate School of Pharmaceutical Sciences, Kyushu University, Higashi-ku, Fukuoka, Japan

**Keywords:** Biotransformation, Cellulolytic enzymes, Isoflavonoid, Natural deep eutectic solvent, *Pueraria mirifica*

## Abstract

The presence of specific gut microflora limits the biotransformation of *Pueraria mirifica* isoflavone (PMI) glycosides into absorbable aglycones, thus limiting their health benefits. Cellulolytic enzyme-assisted extraction (CAE) potentially solves this issue; however, solvent extraction requires recovery of the hydrophobic products. Here, we established the simultaneous transformation and extraction of PMIs using cellulolytic enzymes and natural deep eutectic solvents (NADESs). The NADES compositions were optimized to allow the use of NADESs as CAE media, and the extraction parameters were optimized using response surface methodology (RSM). The optimal conditions were 14.7% (v/v) choline chloride:propylene glycol (1:2 mol ratio, ChCl:PG) at 56.1 °C for the cellulolytic enzyme (262 mU/mL) reaction in which daidzin and genistin were extracted and wholly transformed to their aglycones daidzein and genistein. The extraction of PMIs using ChCl:PG is more efficient than that using conventional solvents; additionally, biocompatible ChCl:PG enhances cellulolytic enzyme activity, catalyzing the transformation of PMIs into compounds with higher estrogenicity and absorbability.

**Supplementary Information:**

The online version contains supplementary material available at 10.1186/s40643-021-00428-9.

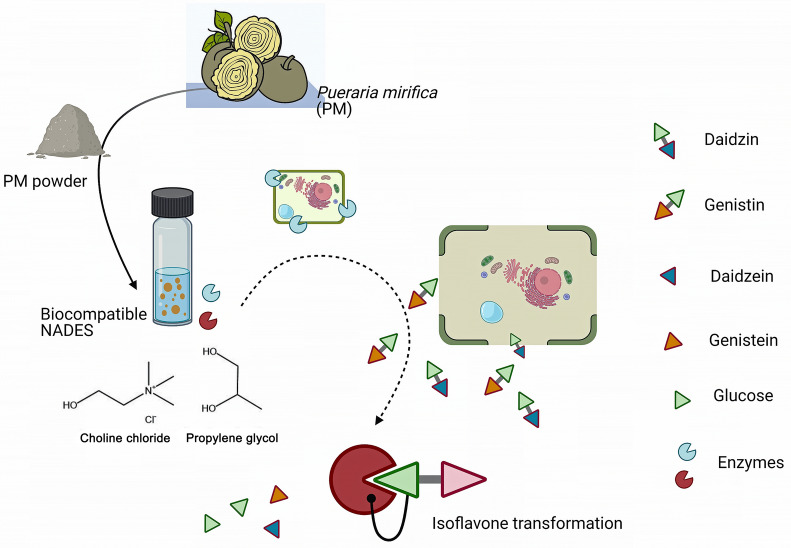

## Introduction

**Fig. 1:**
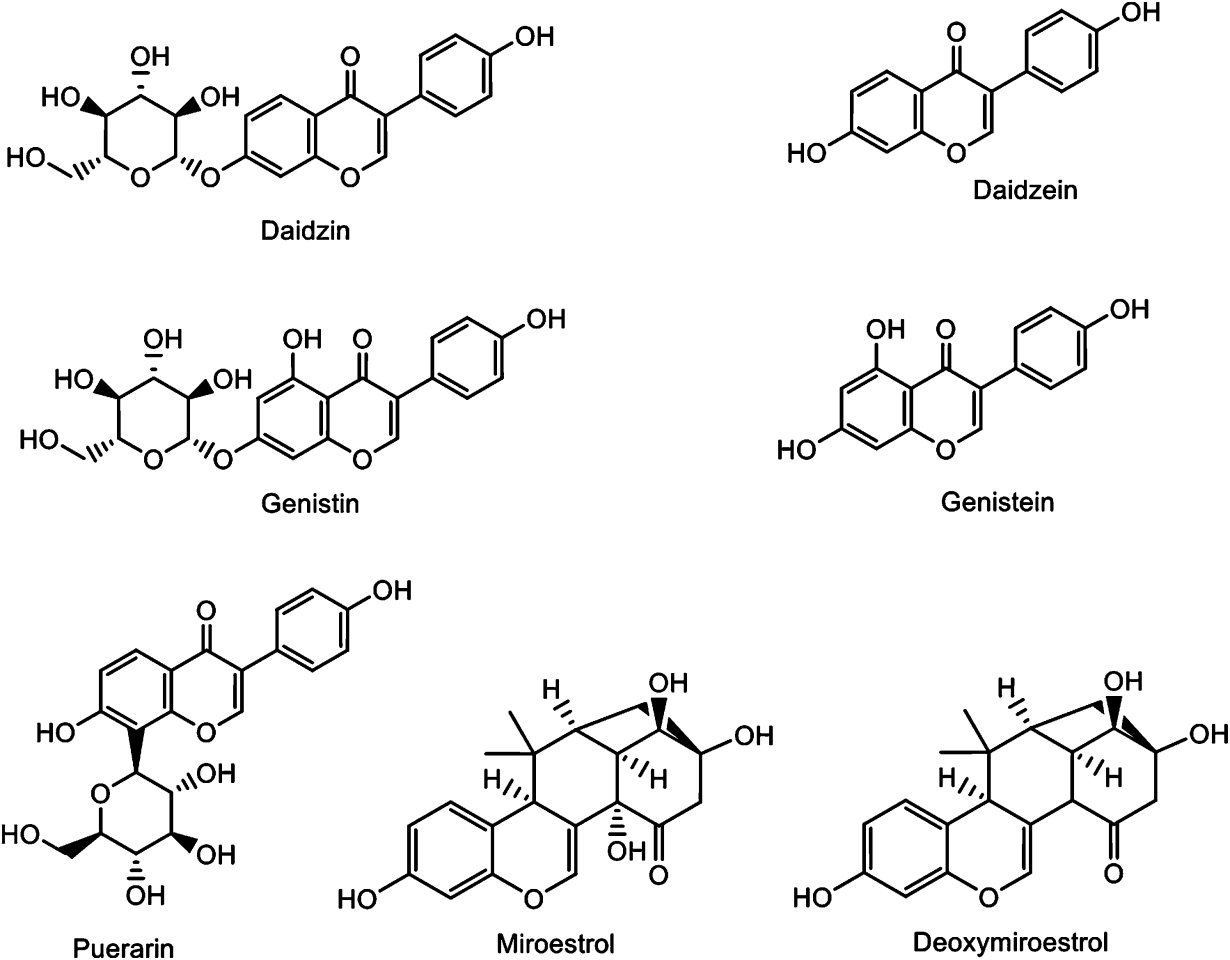
Chemical structures of phytoestrogens, including PUE, DZ, GT, DZe, GTe, MI, and DMI

Phytoestrogens of *Pueraria candollei* var. *mirifica* (Airy Shaw & Suvat.) Niyomdham (PM) have been used as functional health foods. They act as estrogen substances, as proven by clinical studies (Kongkaew et al. 2018). After phytoestrogen consumption, S-equol production in patients is associated with reduced vasomotor symptoms (Newton et al. 2015). The primary factor for S-equol production is the presence of specific intestinal bacteria capable of converting daidzin (DZ) to daidzein (DZe) and DZe to S-equol, consecutively (Setchell et al. 2013). In the human gut environment, strains of *Bifidobacterium* spp. release sugar moieties from isoflavone glycosides. Then, *Bifidobacterium* spp., *Slackia* spp., and other bacteria convert the resultant aglycone into equol (Braune and Blaut 2016). The presence of *Bifidobacterium* is lower in elderly individuals, especially those with certain diseases (Arboleya et al. 2016). Older menopausal women with low levels of isoflavone-metabolizing bacteria may experience little or no benefit from isoflavone phytoestrogens. Isoflavonoids, coumestans, and chromenes are found in the roots of PM (Fig. 1). PM roots contain the potent phytoestrogens miroestrol (MI) and deoxymiroestrol (DMI). The predominant PM isoflavones (PMIs) accumulate in their glycoside forms, such as puerarin (PUE), DZ, and genistin (GT). The bioavailability of DZ and GT is relatively low compared to that of DZe and genistein (GTe) (Okabe et al. 2011). Chemically, acid hydrolysis of isoflavone glycoside produces chemical waste byproducts (Utkina et al. 2004). Isoflavone's bioavailability and biological activity are enhanced when it is fermented with *β*-glycosidase-producing microbes (de Oliveira Silva et al. 2020). Hydrolysis using lactic bacteria is also an effective method, but requires at least 1 day for complete conversion (Pyo et al. 2005). Enzymatic digestion may accelerate the process. The simultaneous transformation of isoflavone glycosides to aglycones during the extraction process may improve the absorption and metabolism of S-equol.

Usually, PMI, MI, and DMI extraction from PM is effective with 95% ethanol maceration (Peerakam et al. 2018). Although bioethanol is a relatively safe and green solvent for herbal extract preparation, ethanol must be removed from the extract, which requires energy, time, and labor. If the resultant extract is further processed for an enzymatic reaction, the solvent must be removed and adjusted to appropriate buffers/pH values. Cellulolytic enzyme-assisted extraction (CAE) of flavonoids from corn husks involves pretreatment with the enzyme in phosphate citrate buffer (pH 5.0). Then, flavonoids are extracted using 80% ethanol at 80 °C for 2 h (Zuorro et al. 2019), and enzyme pretreatment of the plant materials usually involves solvent extraction to recover the flavonoids and isoflavone aglycones (Qadir et al. 2018). Regarding PMI bioconversion, the solubility of the substrates DZ and GT and the products DZe and GTe is low in water and buffer. Thus, biocompatible solvents are crucial for simultaneous extraction/solubilization and enzymatic bioconversion. Natural deep eutectic solvents (NADESs), the ingredients of which are exclusively natural products (nontoxic and environmentally friendly), are useful for phytochemical extraction. Previously, NADESs composed of ChCl:citric acid were used to effectively extract soybean isoflavone (Bajkacz and Adamek 2017). In addition, 1,6-hexanediol:ChCl, acetylcholine chloride:lactic acid, and ChCl:triethylene glycol have been evaluated as appropriate combinations for flavonoid extraction (Mansur et al. 2019). In addition to the solubility capacity, NADESs efficiently respond to microwave heating (González-Rivera et al. 2020), which can shorten the extraction process (Phaisan et al. 2021). NADESs are biologically compatible agents, and ChCl-based NADESs have been shown to enhance the enzyme activity of *β*-glucosidase (Xu et al. 2018). In addition, NADESs enhance the stability and activity of other enzymes, such as CYP79A1 and CYP71E1 (Khodaverdian et al. 2018).

Although NADESs were previously developed to convert partially purified DZ to DZe (Cheng and Zhang 2017), ChCl:ethylene glycol (2:1) was buffered with phosphate buffer. Unbuffered NADESs resulted in less catalytic efficacy. Thus, the development and optimization of processes for the simultaneous extraction of DZ and GT from plant materials and the conversion of these substances to DZe and GTe are required for bioprocessing to enhance the health properties of isoflavones. In this study, cellulolytic enzymes and NADESs were used in these processes. Cellulolytic enzymes are a complex of three different enzymes: endoglucanase (EC 3.2.1.4), exoglucanase (EC 3.2.1.91), and *β*-glucosidase (EC 3.2.1.21) (Singh et al. 2019). This study aims to optimize the NADES composition for *β*-glucosidase activity and then determine the optimal parameters for simultaneous PMI transformation and extraction.

## Material and methods

### Materials

Details of the reagent material and the experimental procedures, including sodium dodecyl sulfate–polyacrylamide gel electrophoresis (SDS-PAGE) analysis and reactivity determination of the cellulolytic enzymes, are described in the Additional file 1. After the enzyme was qualified and quantified for *β*-glucosidase activity, the catalytic reactivity toward glycosides of PMI was evaluated using PM extract.

### Reactivity of enzymes toward PM extract phytochemicals

Because PM extract consists of various constituents, the matrix may negatively impact the catalytic activity of the enzyme toward the target compounds. To ensure that the enzymatic activity in PM extract is retained, the enzymatic reaction of PMIs was evaluated initially using a PM ethanolic extract. The extract was prepared by maceration (500 g of dry PM powder in 2.5 L of 80% ethanol) at room temperature for 2 days. The extract was collected and then concentrated under vacuum using a rotary evaporator and then a lyophilizer. A yield of 37.5 g was obtained. The PM extract was reconstituted with 20% ethanol (100 mg/mL) before enzymatic treatment.

The soluble portion was collected after centrifugation (7800 × *g*, 15 min). The cellulolytic enzyme was prepared at final concentrations of 40, 100, and 500 mU/mL in 50 mM sodium citrate–phosphate buffer at pH 5 (SCP buffer). The reaction was composed of PM extract solution (100 mg/mL, 2.5 mL) and an enzyme solution (2.5 mL). The mixture was then incubated in a shaker at 30 °C. Sample solutions (100 µL) were collected at 0, 5, 10, and 15 min. The reaction was stopped by mixing with 300 µL of 50 mM sodium carbonate buffer (pH 9.6) and immediately diluting with 200 µL of absolute ethanol. The PM extract solution was incubated with enzyme-free SCP buffer as a negative control. The transformation of PM isoflavones was monitored using HPLC. The analytical HPLC method is described in the Additional file 1. When the enzyme was shown to catalyze the transformation of DZ and GT to DZe and GTe, respectively, further experiments were performed to determine the appropriate NADES composition to conserve the enzyme activity.

### Selection of NADES for extraction

The *β*-glucosidase in the cellulolytic enzyme converts DZ to DZe, where DZ is an abundant substrate in the PM extract. The composition and the ratio between hydrogen donor and acceptor of NADES not only influence the enzymatic activity and stability but also affect the target compound solubilization. Thus, the *β*-glucosidase activity of cellulolytic enzymes in the NADESs was determined and used for further optimization of extraction. NADES compositions were designed to retain cellulase activity. Using mole ratios of 2:1, 1:1, and 1:2, the hydrogen acceptor choline chloride (ChCl) was mixed with a hydrogen donor such as propylene glycol (PG) and glycerol (G) and melted at 60 °C. The NADES was then brought to room temperature and diluted to the desired concentration (20, 30, and 40% [v/v]) with deionized water. Then, the *β*-glucosidase activity of the cellulolytic enzymes in the NADES was determined using the method described in the Additional file 1.

ChCl:G and ChCl:PG at a ratio of 1:2 provided high enzyme activity of *β*-glucosidase. Both NADESs were selected for evaluation of their capability to be used for the CAE of PMIs. PM root powder (100 mg) was suspended in the abovementioned NADESs [20 and 40% (v/v), 5 mL] containing 300 mU/mL cellulolytic enzymes. The mixtures were incubated at 30, 50, and 70 °C for 1 h and then microwaved (300 W) for 5 s three times. The solution was isolated after centrifugation at 5000 × *g* for 5 min. The PMIs (PUE, DZ, GT, DZe, and GTe) were determined using HPLC. The microwave-assisted extraction (MAE) condition in this research was selected based on the optimization explained in the Additional file 1.

### Response surface methodology (RSM) for extraction optimization

Because the NADES ChCl:PG (1:2) provides an environment for high *β*-glucosidase activity and extraction capacity of enzymatic products (DZe and GTe), the effects of the extraction parameters, including ChCl:PG concentration, temperature, enzyme concentration, and incubation time, were further optimized. The CAE of PMI biotransformation and extraction was studied when varying the ChCl:PG concentration. The PM root powder (100 mg) was suspended in various concentrations of ChCl:PG (5–50% (v/v), 5 mL) containing 300 mU/mL cellulolytic enzymes. The mixture was incubated at 50 °C for 1 h. The incubation temperature was studied in the range of 40–80 °C. The PM root powder (100 mg) was incubated in 20% ChCl:PG (5 mL) containing 300 mU/mL cellulolytic enzymes for 1 h. At the optimal ChCl:PG concentration and temperature, the enzyme concentration (100–600 mU/mL) was optimized. Different concentrations of cellulolytic enzymes were prepared in 20% ChCl:PG. The PM root powders (100 mg) were suspended in the enzyme solution and then incubated at 60 °C for 1 h. Then, the duration of the extraction was varied. The PM root powders (100 mg) were suspended in 200 mU/mL cellulolytic enzymes in 20% ChCl:PG and then incubated at 60 °C for 30–150 min. After treatment of PM powders according to the above-designed procedure, a microwave was applied, and the PMIs in the extract were determined.

The interactions between ChCl:PG concentration, temperature, and enzyme concentration impact the extraction efficacy. For example, the temperature impacts NADES viscosity and enzyme activity, and the concentration and viscosity of NADES influence the enzymatic activity. Regarding the single-parameter optimization, the optimal conditions consisted of 20% ChCl:PG, 60 °C incubation temperature, and 200 mU/mL cellulolytic enzymes, and under these conditions, DZ and GT were completely converted into their aglycones within 1 h. RSM was applied to determine the optimal conditions based on factor interactions. A statistical experiment using the Box–Behnken design (BBD) of RSM was applied. The experiment was designed with 17 treatments, which included five repeats of the center point, with the aim of identifying optimal conditions after the interactions between factors were addressed. Three levels of independent factors, namely, the ChCl:PG concentration (*X*_*1*_) (5, 20, and 35%), incubation temperature (*X*_*2*_) (30, 60, and 90 °C), and cellulolytic enzyme concentration (*X*_*3*_) (20, 200, and 380 mU/mL), were evaluated (Additional file 1: Table S3). The yields of DZe (*Y*_*1*_) and GTe (*Y*_*2*_) were dependent factors. The interaction between the independent parameters influencing each response was analyzed in a random order to determine the fittest model. All statistical analyses were performed by means of Design Expert 13. The statistical significance of the model obtained from BBD was investigated by analysis of variance (ANOVA), with significance determined at *p*-values less than 0.05.

### Efficacy of the extraction methods

The MI and DMI are the potent phytoestrogens of PM, and the CAE was evaluated to ensure recovery of the compounds. Based on RSM, the optimal condition was selected for this experiment. The extraction efficiency of CAE for PMIs, MI, and DMI was compared with conventional solvents, including water, 50% ethanol in water, 80% ethanol in water, and absolute ethanol. PM (5 g) was extracted for 1 h with 250 mL of 20% ChCl:PG containing 200 mU/mL cellulolytic enzymes. The PM phytochemicals were recovered when all the extract solutions were subjected to octadecylsilane (C18) resin (30 mL resin volume), which was equilibrated with 5% ethanol. The C18 resin was washed using five resin volumes of water (150 mL). After that, the bound PM phytochemicals were eluted using 80% (v/v) ethanol in water. In addition, PM was extracted using 20% ChCl:PG without cellulolytic enzymes and the abovementioned conventional solvents; the extracts were treated using the same procedure. The extracts were vacuum evaporated (40 °C) and lyophilized. The yields of PMIs, MI, and DMI were determined using HPLC and an indirect competitive enzyme-linked immunosorbent assay (icELISA), and the procedures are described in the Additional file 1.

### Determination of PM phytochemicals

The PMIs, including PUE, DZ, GT, DZe, and GTe, were determined using HPLC with some modifications (Yusakul et al. 2020). The MI and DMI yields were determined using icELISA (Yusakul et al. 2018). The detailed procedures are described in the Additional file 1.

### Statistical analysis

All the experiments were performed in triplicate. The data are presented as the mean ± standard deviation (SD). Statistically significant differences were determined by ANOVA using SPSS ver. 26.

## Results and discussion

### Catalytic reactivity of enzymes toward PMIs

Cellulolytic enzymes convert DZ and GT to DZe and GTe in a concentration-dependent manner. Cellulolytic enzymes at all concentrations converted DZ into DZe. GTe was not observed at a low cellulolytic enzyme concentration (40 mU/mL). However, the conversion of GT to GTe was observed with 100 and 500 mU/mL cellulolytic enzymes. Higher enzyme concentrations accelerated DZe and GTe production (Additional file 1: Fig. S4, Table S4). GTe production was less than DZe production because the GT content of PM was less than that of DZ. The enzymatic rate increased with increasing substrate concentration (Liu 2017). Cellulolytic enzymes did not change the level of PUE in the reaction mixture because PUE contains a C-glycosidic bond. *β*-Glucosidase is reactive toward the *β*-O-glycosidic bonds of DZ and GT (Chen et al. 2016). Thus, the enzymes cannot cleave PUE with C-glycosidic bonds. The cleavage of PUE to produce DZe is mediated by enzymes obtained from *Dorea* spp. (Nakamura et al. 2020). In addition, PUE can be hydrolyzed to DZe using an ionic liquid coupled with microwave irradiation (Wang et al. 2018).

### Development of NADESs as enzymatic media

**Fig. 2:**
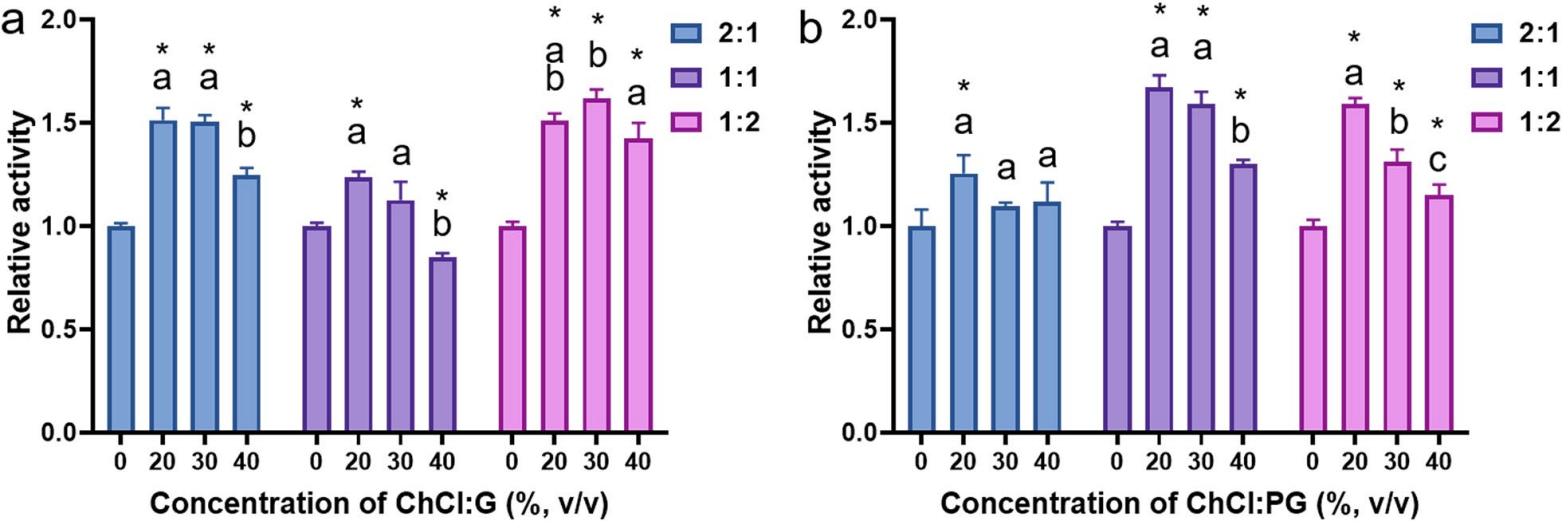
The effects of NADES compositions and concentrations on the *β*-glucosidase activity of cellulolytic enzymes. The relative activity is the ratio between the cellulolytic enzyme activity in the NADES ChCl:G (**a)** and ChCl:PG (**b)** and that in the control buffer (50 mM sodium-citrate phosphate buffer, pH 5). The * indicates a significant difference in cellulolytic enzyme activity in the NADESs compared to the activity in the control buffer. The same and different letters indicate nonsignificant and significant differences in relative activity, respectively, when the data are compared between 20 and 30% concentrations for each NADES composition. Statistical significance was determined by one-way ANOVA, followed by LSD (*p* < 0.01)

The activity of cellulolytic enzymes in all NADESs was compared with the activity in SCP buffer. The results demonstrated the relative activity, which was the ratio of the catalytic activity of cellulolytic enzymes in NADESs to the activity in the buffer (Fig. 2). When the same enzyme concentration was used in the experiment, the relative activity in almost all ChCl:G NADESs was higher than that in SCP buffer (0.85–1.63 relative activity). When the concentration of ChCl:G was 40%, the enzyme activity decreased. NADESs are quite viscous, which decreases enzyme activity. ChCl:G (1:1) at a concentration of 40% suppressed cellulolytic enzyme activity compared to cellulolytic enzyme activity in SCP buffer (0.85 relative activity). Interestingly, all ratios and concentrations (20–40%, v/v) of ChCl:PG resulted in high activity (1.10–1.67 relative activity). The composition of ChCl:PG has also been reported to improve *β*-glucosidase compared with ChCl:G, and NADESs have been shown to preserve enzyme stability (Xu et al. 2018). Both the composition and ratio of NADESs influence enzyme stability and activity because free components can permeate the enzyme structure and interfere with hydrogen bonding. The appropriate NADES achieves a balance between the individual components and prevents their diffusion into the protein core (Xu et al. 2018). NADESs, as enzymatic media, preserve both the enzyme function and solubility of the target compound. NADES-based reactions are safe for human food processing because of the reduced possibility of chemical byproduct contamination in the products, and NADESs are not toxic.

### Screening of NADESs for cellulolytic enzyme-assisted extraction of PMI

The NADESs, which supported the high activity of ChCl:G (1:2) and ChCl:PG (1:2), were further compared for the CAE of PMIs. The effect of the NADES concentration and temperature on the extraction and bioconversion of PMIs was investigated. The yields of PUE extracted by the abovementioned NADESs were in the range of 1.01–1.23 × 10^3^ μg/g and tended to decrease with increasing temperature (Additional file 1: Fig. S5). The yields of DZ decreased with increasing DZe yield because DZ was converted into DZe by *β*-glucosidase activity. The results indicated that ChCl:PG (1:2 mol ratio, 20%, v/v) provided the highest DZe yield (400 µg/g), and the enzyme activity tended to increase with increasing temperature. The DZe extraction yields by 20% ChCl:PG were higher than those with 40% ChCl:PG because the former provided higher cellulolytic enzyme activity (Fig. 2). The extraction yields of GT and GTe were in the ranges of 41.0–66.8 µg/g and 59.3–67.5 µg/g, respectively, among the tested ChCl:G concentrations and temperatures. ChCl:PG produced similar yields in the ranges of 40.9–66.7 and 51.5–68.8 µg/g GT and GTe, respectively. Thus, ChCl:PG was appropriate for consequent development.

ChCl:G (40%) provided a high yield of DZe product at 70 °C (370 µg/g), which implies that the higher concentration of ChCl:G preserved the enzyme activity at that temperature. The NADESs prevented the enzyme degradation induced by high temperature, presumably because of the formation of an ionic supramolecular net around the enzyme molecules (Xu et al. 2018). The ChCl:PG NADES-based CAE produced a higher yield of DZe at elevated temperatures. ChCl:PG maintained the enzyme activity and resulted in high DZe yields in a temperature-dependent manner. Temperature directly influences enzyme activity and stability, and elevated temperature decreases the viscosity of NADESs. A lower-viscosity solvent increases the extraction efficacy (Fernandez et al. 2018). ChCl:PG (1:2), which produced a high yield of PMIs in the selected temperature range, was selected for further optimization. ChCl:PG is less viscous than ChCl with sugars, such as glucose and sucrose, resulting in ChCl:PG maintaining higher *β*-glucosidase activity (Xu et al. 2018). In conclusion, NADESs help preserve the native conformation of the enzyme structure.

### Optimization of cellulolytic enzyme-assisted extraction of PMI

#### Effect of NADES concentration

**Fig. 3:**
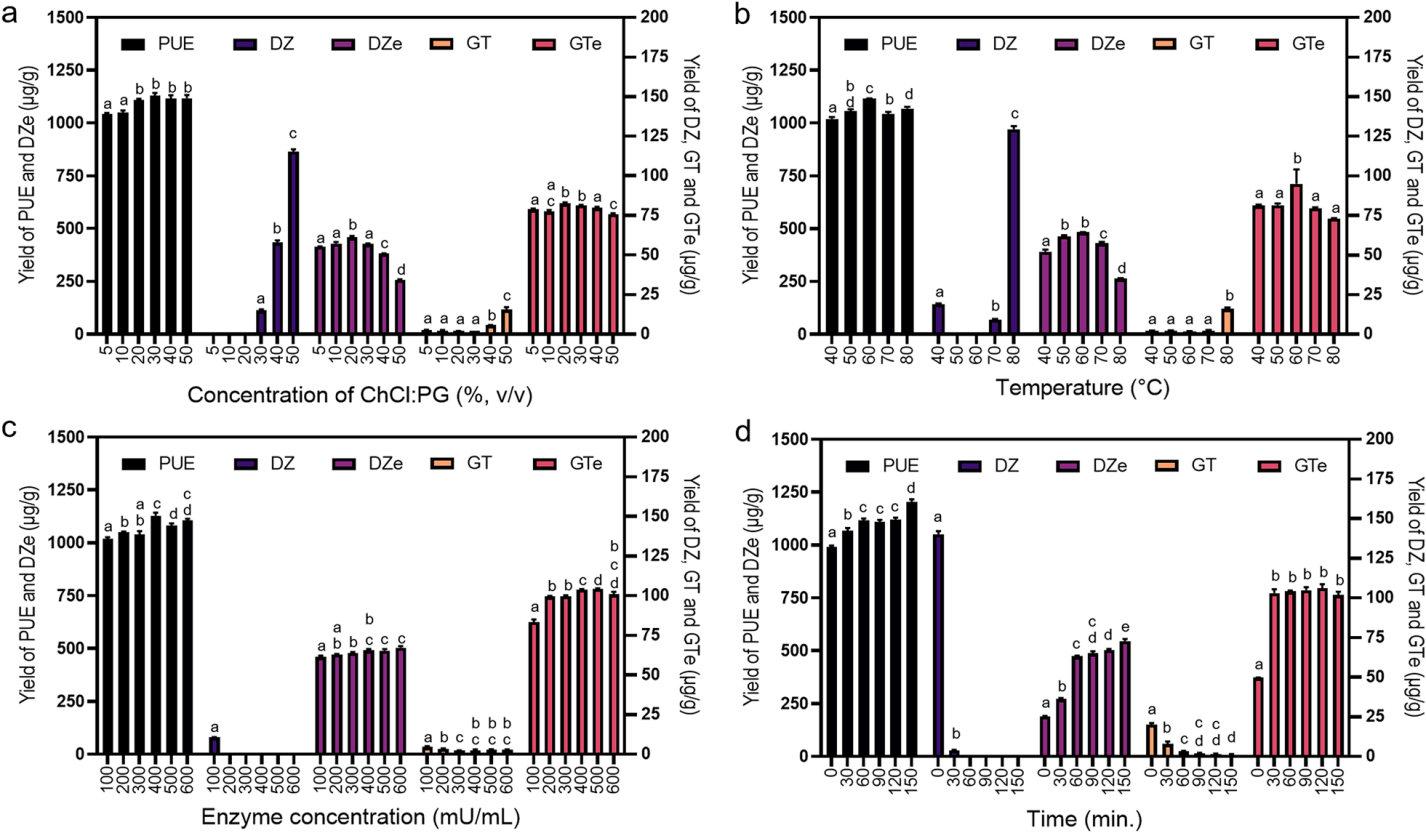
The effect of each extraction parameter on the extractive yields of PMIs (PUE, DZ, GT, DZe, and GTe), where the concentration of ChCl:PG (1:2) (**a**), temperature (**b**), concentration of cellulolytic enzymes (**c**), and duration of extraction (**d**) were investigated. The same and different letters indicate nonsignificant and significant differences in extractive yields of each compound, respectively, when the data are the levels of each parameter. Statistical significance was determined by one-way ANOVA, followed by LSD (*p* < 0.01)

The yields of PUE, DZe, and GTe increased to maximum values in the presence of 20% ChCl:PG (1:2). However, the yields of DZe and GTe were slightly reduced at ChCl:PG (1:2) concentrations higher than 20% v/v, as shown in Fig. 3a (Additional file 1: Table S5). This is due to the increased viscosity of the solution, which decreased the enzyme activity and extraction efficacy. At a high concentration of ChCl:PG (1:2), the extractive PMIs existed in the form of glycosides DZ and GT. An experiment with *β*-D-glucosidase indicated that the enzyme activity decreased when the concentration of NADESs (ChCl: ethylene glycol and ChCl: G NADES) was over 30% (Cheng and Zhang 2017). The high NADES viscosity limited the mass transfer of substrate extraction and its transfer to the enzyme binding site. The viscosity of the medium decreased molecular motion, inhibiting catalysis in motile enzymes (Uribe and Sampedro 2003). Since a 20% v/v ChCl:PG (1:2) concentration provided extraction efficacy and maintained the enzyme activity, it was chosen for condition optimization.

#### Effect of temperature

The reaction solution's temperature, where NADESs were used as a medium, affected the medium viscosity, mass transfer, enzyme activity, enzyme stability, and target compound solubility. The temperature of the incubating shaker was varied from 40 to 80 °C. The extraction efficacy of PUE, DZe, and GTe was enhanced when the incubation shaker temperature increased from 40 to 60 °C. After that, the responses of the aglycone were decreased (Fig. 3b, Additional file 1: Table S6). The viscosity of glycols and ChCl-based NADESs was markedly decreased when the temperature increased from 20 to 60 °C (Gajardo-Parra et al. 2020). The activity of *β*-glucosidase from *Trichoderma reesei* is optimal at temperatures between 60 and 70 °C (Chen et al. 1992). A temperature of 60 °C is optimal for low-viscosity NADESs and enzyme activity, resulting in high yields of the target DZe and GTe. This temperature was selected for the further optimization of other parameters.

#### Effect of the cellulolytic enzyme concentration

Enzyme concentrations play an essential role in the extraction of substrates and the conversion of substrates to products. The endoglucanase activity was expected to cleave cellulose, which then allowed the NADES to penetrate the cell and dissolve the substrates, which were then converted by *β*-glucosidase action. Cellulase-assisted cell wall disruption enhanced the extraction of Z-ligustilide from Radix *Angelica sinensis* (Zhang et al. 2017) and resveratrol from *Polygonum cuspidatum* (Wang et al. 2019). The extraction yields of PUE, DZe, and GTe reached a maximum at 400 mU/mL enzyme (1.13 ± 0.014 × 10^3^, 491 ± 6.69, and 104 ± 0.31 µg/g). A greater enzyme concentration did not significantly improve the yield. However, at 200 mU/mL, DZ and GT were almost completely transformed to DZe and GTe (Fig. 3c, Additional file 1: Table S7), with yields of 473 ± 1.44 and 99.4 ± 0.52, respectively. Therefore, the minimal enzyme concentration of 200 mU/mL was selected because it appeared sufficient.

#### Effect of the reaction time

The reaction time is one of the essential parameters that influences bioactive compound responses, probably because this is the time allowed for the reaction between the enzyme and the target compounds. The reaction time was varied from 30 to 150 min at intervals of 30 min. At the initial time, the yield of PUE was relatively low, and DZ and GT remained untransformed. The results imply that ChCl:PG (1:2) NADESs can extract substrates (DZ and GT). With a longer incubation time, DZ and GT were converted to DZe and GTe; DZ was undetectable at 60 min. The yields of DZe and GTe increased as the reaction time increased from 30 to 60 min (Fig. 3d, Additional file 1: Table S8). Hence, a reaction time of 60 min was used because this is the shortest reaction time that provided high yields of DZe and GTe. Enzymatic conversion consumes less time than the process of microbial fermentation (Pyo et al. 2005).

#### Cellulolytic enzyme-assisted extraction via RSM

The BBD of RSM was performed to identify the best conditions. The responses of the designed experiment are shown in Table S9. ANOVA was used to inform a reduced quadratic model that fit the responses, with F-value results of 28.49 for DZe (*Y*_*1*_) and 24.07 for GTe (*Y*_*2*_) and *p*-values of < 0.0001 for *Y*_*1*_ and *Y*_*2*_. The factors *X*_*1*_, *X*_*2*_, *X*_*3*_, *X*_*1*_*X*_*2*_, *X*_*1*_*X*_*3*_, *X*_*2*_^*2*^, and *X*_*3*_^*2*^ for DZe (*Y*_*1*_) and *X*_*2*_, *X*_*1*_*X*_*2*_, and *X*_*2*_^*2*^ for GTe (*Y*_*2*_) significantly influenced the model, as shown in Table 1. The linear regression values (adjusted *R*^*2*^) were 0.9232 and 0.8556 for *Y*_*1*_ and *Y*_*2*_, respectively. The predicted *R*^2^ is in reasonable agreement with the adjusted *R*^2^, with a difference of less than 0.2, indicating that the models were reliable and precise to predict the response. Moreover, the lack of fit values were obtained to be an *F*-value of 3.39 (*p* = 0.1301) for *Y*_*1*_ and an *F*-value of 4.41 (*p* = 0.0841) for *Y*_*2*_, indicating that the responses fitted with the model. The regressions between the independent and dependent variables of *Y*_*1*_ and *Y*_*2*_ are shown in the coded equations below:

1 $$Y_{1} = 485.33 - 23.96X_{1} - 31.91X_{2} + 31.05X_{3} + 36.49X_{1} X_{2} + 30.08X_{1} X_{3} - 177.38X_{2}^{2} - 40.28X_{3}^{2} ,$$

2 $$Y_{2} = { 91}.{71 } - \, 0.{5269}X_{1} {-}{ 2}.{42}X_{2} + { 1}.{47}X_{1} X_{2} {-}{ 5}.{48}X_{2}^{2} .$$

**Table 1:** ANOVA data of regression parameters of the Box–Behnken design experiment

Source of variation	*Y*_*1*_			*Y*_*2*_		
	Sum of squares	*F*-value	*P*-value probability	Sum of squares	*F*-value	*P*-value probability
Model	1.729E + 05	28.49	< 0.0001	184.89	24.07	< 0.0001
*X*_*1*_	4592.58	5.30	0.0469	2.22	1.19	0.2974
*X*_*2*_	8147.75	9.40	0.0136	46.93	25.08	0.0003
*X*_*3*_	7713.23	8.90	0.0154	–	–	–
*X*_*1*_*X*_*2*_	5327.14	6.14	0.0351	8.67	4.63	0.0524
*X*_*2*_*X*_*3*_	–	–	–	–	–	–
*X*_*1*_*X*_*3*_	3620.34	4.18	0.0714	–	–	–
*X*_*1*_^*2*^	–	–	–	–	–	–
*X*_*2*_^*2*^	1.328E + 05	153.22	< 0.0001	127.06	67.89	< 0.0001
*X*_*3*_^*2*^	6849.18	7.90	0.0204	–	–	–
Residual	7803.08			22.46		
Lack of fit	6313.69	3.39	0.1301	20.17	4.41	0.0841
Pure error	1489.39			2.29		
Cor total	1.807E + 05			207.34		
Std Dev	29.45			1.37		
Mean	382.90			89.14		
C.V. (%)	7.69			1.53		
PRESS	47,647.95			52.07		
*R*^2^	0.9568			0.8917		
Adjusted *R*^2^	0.9232			0.8556		
Predicted *R*^2^	0.7363			0.7489		

**Fig. 4:**
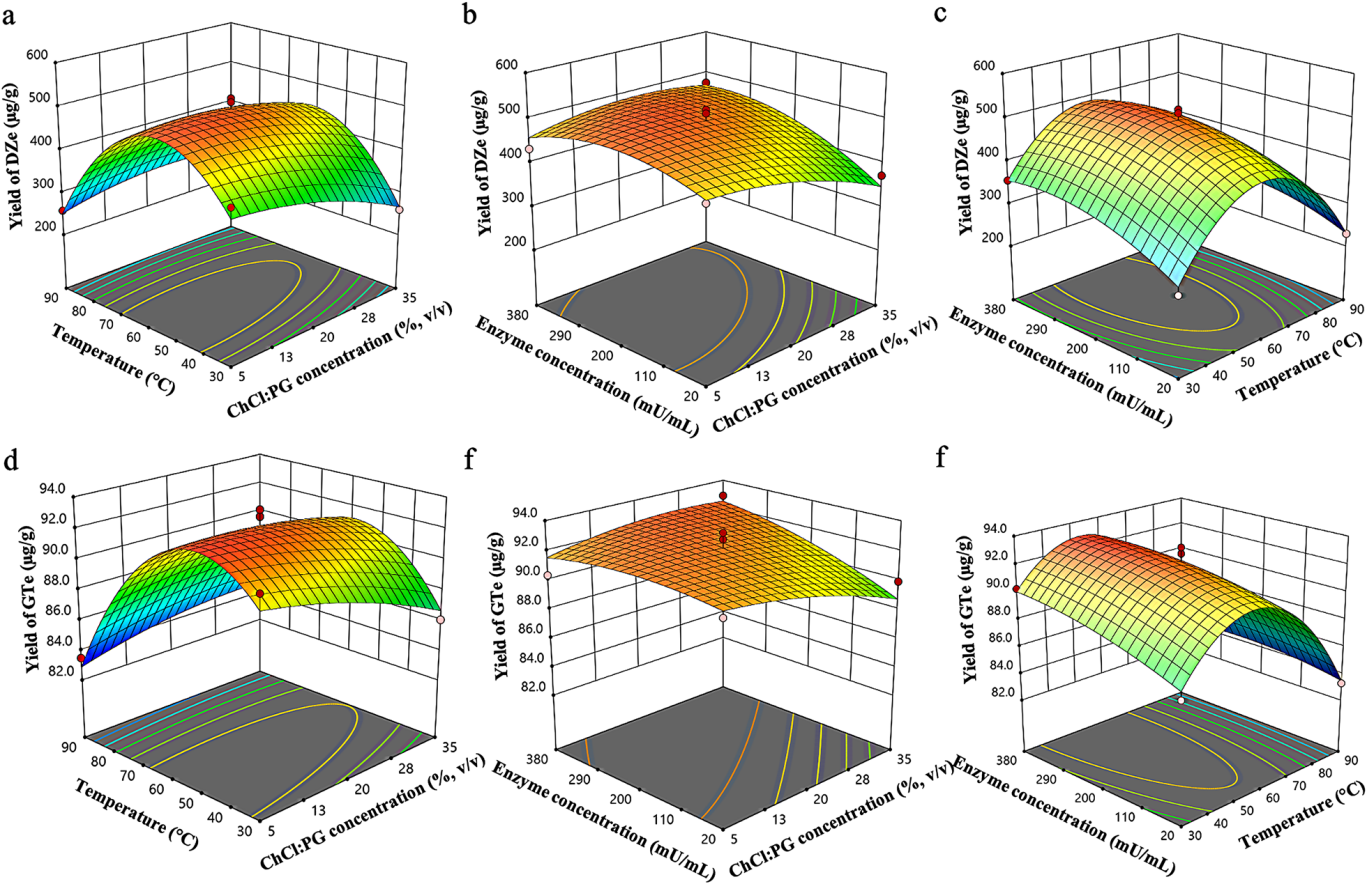
3D plots of the responses of DZe (*Y*_*1*_) (**a**–**c**) and GTe (*Y*_*2*_) (**d**–**f**), demonstrating the effect of interaction between independent factors, including NADES concentration (*X*_*1*_), temperature (*X*_*2*_), and concentration of cellulolytic enzymes (*X*_*3*_)

Based on the interaction between the factors, the optimal conditions for *Y*_*1*_ were defined as 14.7% (v/v) ChCl:PG (1:2), 56 °C incubation temperature, and 262 mU/mL cellulolytic enzymes. The optimal conditions for *Y*_*2*_ were 14.7% (v/v) ChCl:PG (1:2), 47 °C incubation temperature, and 250 mU/mL cellulolytic enzymes. Three-dimensional (3D) responses were illustrated to determine the interaction effect of independent parameters (*X*_*1*_, *X*_*2*,_ and *X*_*3*_) on the responses of *Y*_*1*_ and *Y*_*2*_, as presented in Fig. 4. For the *Y*_*1*_ response, the optimal temperature of CAE was 50–61 °C, and at higher concentrations of ChCl:PG (1:2), the elevated temperature maintained a suitable medium viscosity for the enzyme reaction. In the same pattern, a higher concentration of ChCl:PG (1:2) needed a higher enzyme concentration to achieve a high response because the viscosity decreased the enzyme activity. However, at the optimal temperature, the enzyme concentration (221–330 mU/mL) did not strongly impact the response. For the *Y*_*2*_ response, the factor interaction produced a trend similar to that of the *Y*_*1*_ responses because the same enzyme produced DZe and GTe. The chemical structures of these products are quite similar as well. Overall, the optimal temperature limit in the specific range slightly increased when the concentration of ChCl:PG (1:2) increased. However, the enzyme concentration must be greatly increased to achieve a high response.

The advantages of RSM included improving the yields and reducing the process cost. Single-parameter optimization provided a high response at 20% (v/v) ChCl:PG, 60 °C, and 400 mU/mL cellulase, and DZe was produced at the levels of 459, 483, and 491 µg/mg. The optimal conditions obtained via RSM were 14.7%, 56 °C, and 262 mU/mL, which produced similar yields to those of the single-parameter optimization. However, the cost of solvent and enzyme could be reduced. In addition, the energy for temperature could be lowered.

The established reduced quadratic models for the prediction of *Y*_*1*_ and *Y*_*2*_ were verified with the optimal condition of *Y*_*1*_ because *Y*_*1*_ is a major constituent compared to *Y*_*2*_. *X*_*1*_, *X*_*2*_, and *X*_*3*_ values of 14.7%, 56 °C, and 262 mU/mL were used. The *Y*_*1*_ and *Y*_*2*_ yields of 499 and 92 µg/mg were expected from the models, with 95% prediction intervals (PIs) of 42–571 and 89–95 µg/mg, respectively. The experimental yields were 514 ± 1.98 (*Y*_*1*_) and 109 ± 1.53 (*Y*_*2*_) µg/mg, which corresponded to a 97 and 84% prediction accuracy, respectively. Thus, the model verification informs the reliability of *Y*_*1*_. The process should be additionally optimized for *Y*_*2*_, and other factors, for example, the liquid-to-solid ratio and particle size of the PM root powder, should be included to obtain the fitted model.

### Efficacy of CAE

The efficacy of CAE was compared with that of the extraction without cellulolytic enzymes. In addition, extraction using conventional solvents, namely, water, 50% ethanol, 80% ethanol, and absolute ethanol, was performed. When ChCl:PG NADES (1:2, without cellulolytic enzymes) was used to extract PM phytochemicals, the yield of all PMIs was significantly higher than those extracted with conventional organic solvents (Table 2). However, ChCl:PG (1:2, without cellulolytic enzymes) exhibited less effectiveness than absolute ethanol in extracting the potent phytoestrogens MI and DMI. Cellulolytic enzymes assisted the transformation of DZ and GT to DZe and GTe, respectively. CAE produced a nonsignificant difference in the yield of total MI and DMI as compared to those produced by absolute ethanol. Thus, the process enhanced the DZe and GTe contents without loss of potent PM phytoestrogens. The enzyme enhanced the yields of MI and DMI. ChCl:PG (1:2) provided the extraction capability and biocompatibility for an enzyme reaction. PM phytoestrogen extraction was as effective as extraction with 95% ethanol, which maximizes the recovery of both predominant PMIs and potent MI and DMI (Peerakam et al. 2018). The resultant extracts with 95% ethanol comprise predominantly DZ and GT rather than their aglycones after 7 days of maceration. Ultrasonically assisted extraction of isoflavonoids from *Pueraria lobata* (Willd.) Ohwi required 49 min using 71.35% ethanol (Xu et al. 2007). The CAE of PM phytochemicals is effective and environmentally friendly. The transformation of DZ and GT to DZe and GTe enhanced the health benefits of the isoflavones. The DZe flavor is associated with equol production status and equol functions in menopause relief, cardiovascular protection, and osteoporotic prevention (Daily et al. 2019; Mayo et al. 2019). The bioavailability of DZ and GT is much less than that of DZe and GTe (Izumi et al. 2000; Setchell et al. 2002). DZe and GTe provide greater health benefits than DZ and GT, such as LDL oxidation (Lee et al. 2005), inhibition of human breast cancer cells (Peterson and Barnes 1991), and inhibition of bone loss (Nirmala et al. 2019).

**Table 2:** Comparison of the efficacy of solvents for PM phytochemical extraction

Solvents	Yields (mg/g extract)					Total MI and DMI (mg/g extract)
	PUE	DZ	GT	DZe	GTe	
ChCl:PG (1:2) with cellulolytic enzymes	138 ± 2.42^a^	0.919 ± 0.33^a^	0.669 ± 0.03^a^	69.1 ± 1.17^a^	3.13 ± 0.07	9.11 ± 0.82^a^
ChCl:PG (1:2) without cellulolytic enzymes	106 ± 7.48^b^	18.8 ± 1.57^b^	13.2 ± 1.32^b^	18.5 ± 1.84^b^	ND	6.44 ± 0.39^b^
Water	26.1 ± 0.26^c^	4.61 ± 0.05^c^	1.31 ± 0.02^a^	4.36 ± 0.06^c^	ND	0.531 ± 0.104^c^
50% ethanol	8.72 ± 0.15^d^	0.754 ± 0.02^c^	0.684 ± 0.03^a^	1.97 ± 0.05^d^	ND	1.18 ± 0.10^c^
80% ethanol	14.9 ± 0.20^d^	3.03 ± 0.04^c^	1.60 ± 0.01^a^	2.34 ± 0.08^c, d^	ND	1.87 ± 0.12^d^
Absolute ethanol	10.5 ± 0.19^d^	2.01 ± 0.02^c^	1.16 ± 0.05^a^	1.47 ± 0.04^d^	ND	9.05 ± 0.84^a^

The same and different letters indicate nonsignificant and significant differences in extractive yields, respectively. Statistical significance was determined by one-way ANOVA, followed by LSD (*p* < 0.05). *ND* not detectable

## Conclusion

An NADES composed of ChCl:PG (1:2) provided a high *β*-glucosidase activity of cellulolytic enzymes, which was superior to the enzyme activity in SCP buffer. The efficacy of ChCl:PG (1:2) for PMI extraction is comparable to that of absolute ethanol. The optimal conditions for the simultaneous extraction and transformation of PM isoflavones were 14.7% (v/v) ChCl:PG (1:2), 56 °C incubation temperature, and 262 mU/mL cellulolytic enzymes. The incubation time was 1 h. Overall, the procedures are rapid, simple, efficient, and safe. This method has a high potential to be used for other flavonoid-containing food plants, such as soybean, *P. lobata*, and other legumes. The use of cellulolytic enzymes coupled with NADESs is a sustainable process for the health food industry.

### Supplementary Information

**Additional file 1.** 1) Materials, 2) Analysis and quantification of cellulolytic enzymes, 3) Analysis of enzyme activity, 4) Determination of isoflavonoids using high-performance liquid chromatography coupled UV–Vis detector, 5) Determination of miroestrol and deoxymiroestrol using indirect competitive enzyme-linked immunosorbent assay (icELISA), and 6) optimization of microwave-assisted extraction. **Fig. S1.** SDS-PAGE of the cellulolytic enzyme from *Trichoderma reesei*. Lane 1 shows protein molecular mass markers, and the other lane (lane 2) shows cellulolytic enzymes. **Fig. S2.** Chromatograms of authentic compounds (**a**), including PUE, DZ, GT, DZe, and GTe, and the compounds extracted using 80% ethanol (**b**), ChCl:PG without cellulolytic enzymes (**c**), and ChCl:PG with cellulolytic enzymes (**d**). **Fig. S3.** The calibration curves of miroestrol via icELISA. **Fig. S4.** The catalytic activity of cellulolytic enzymes at 40 mU/mL (**b**), 100 mU/mL (**c**), and 500 mU/mL (**d**) with PMIs (PUE, DZ, GT, DZe, and GTe), where control (**a**) was performed without the enzyme. The *indicates a significant difference compared to the concentration at the initial time (*p* < 0.05). **Fig. S5.** The extraction and biotransformation of PMIs using cellulolytic enzymes with different NADESs, including 20% ChCl:G (**a**), 40% ChCl:G (**b**), 20% ChCl:PG (**c**), and 40% ChCl:PG (**d**), in which the reactions were conducted in the temperature range of 30, 50, and 70 °C. The same and different letters indicate nonsignificant and significant differences in the extractive yield of each compound between the temperature treatments, respectively. Statistical significance was determined by one-way ANOVA, followed by LSD (*p* < 0.01). **Table S1.** The effect of microwave power on the yields of PMIs extracted by MAE. **Table S2** The effect of irradiation time on the yields of PMIs extracted by MAE. **Table S3.** The experimental variable factors of the BBD for the extraction of PMIs from PM. **Table S4.** The rate of daidzein production using cellulolytic enzymes. **Table S5.** The effect of ChCl:PG concentration on the PMI extraction and transformation efficiency. **Table S6.** The effect of temperature on the PMI extraction and transformation efficiency. **Table S7.** The effect of cellulolytic enzyme concentration on PMI extraction and transformation efficiency. **Table S8.** The effect of time on the PMI extraction and transformation efficiency. **Table S9.** The experimental design matrix of BBD and their results of daidzein (*Y*_*1*_) and genistein (*Y*_*2*_).

## Data Availability

All data and materials are available in the main text and supporting information.

## Abbreviations

PM: *Pueraria candollei* var. *mirifica* (Airy Shaw & Suvat.) Niyomdham
PMI: *Pueraria mirifica* Isoflavone
CAE: Cellulolytic enzyme-assisted extraction
DZ: Daidzin
DZe: Daidzein
MI: Miroestrol
DMI: Deoxymiroestrol
PUE: Puerarin
GT: Genistin
GTe: Genistein
SDS-PAGE: Sodium dodecyl sulfate–polyacrylamide gel electrophoresis (SDS-PAGE)
ChCl: Choline chloride
PG: Propylene glycol
G: Glycerol
MAE: Microwave-assisted extraction
BBD: Box–Behnken design
NADES: Natural deep eutectic solvent
RSM: Response surface methodology
