# Correction to: Biocompatible natural deep eutectic solvent‑based extraction and cellulolytic enzyme‑mediated transformation of *Pueraria mirifica* isoflavones: a sustainable approach for increasing health‑bioactive constituents

**DOI:** 10.1186/s40643-021-00437-8

**Published:** 2021-09-02

**Authors:** Fonthip Makkliang, Boondaree Siriwarin, Gorawit Yusakul, Suppalak Phaisan, Attapon Sakdamas, Natthapon Chuphol, Waraporn Putalun, Seiichi Sakamoto

**Affiliations:** 1 School of Languages and General Education, Walailak University, Nakhon Si Thammarat, Thailand; 2 Faculty of Pharmaceutical Sciences, Huachiew Chalermprakiet University, Samut Prakan, Thailand; 3 School of Pharmacy, Walailak University, Nakhon Si Thammarat, 80160 Thailand; 4 Biomass and Oil Palm Center of Excellence, Walailak University, Nakhon Si Thammarat, Thailand; 5 Faculty of Pharmaceutical Sciences, Prince of Songkla University, Songkhla, Thailand; 6 Faculty of Pharmaceutical Sciences, Khon Kaen University, Khon Kaen, Thailand; 7 Graduate School of Pharmaceutical Sciences, Kyushu University, Higashi‑ku, Fukuoka, Japan

## Correction to: Bioresour Bioprocess (2021) 8:76 https://doi.org/10.1186/s40643-021-00428-9

In the original publication of the article (Makkliang et al. 2020) the reference “Phaisan et al. 2020” was incorrect and should have been mentioned as “Yusakul et al. 2020”. The corrected reference is given in this Correction article.

- Yusakul G, Juengsanguanpornsuk W, Sritularak B, Phaisan S, Juengwatanatrakul T, Putalun W (2020) (+)-7-*O*-Methylisomiroestrol, a new chromene phytoestrogen from the *Pueraria candollei* var. mirifica root. Nat Prod Res. 10.1080/14786419.2020.1727473

The original article has been corrected.
