# Supplementary material for: Biocompatible natural deep eutectic solvent-based extraction and cellulolytic enzyme-mediated transformation of Pueraria mirifica isoflavones: a sustainable approach for increasing health-bioactive constituents
Source: Bioresour Bioprocess. 2021 Aug 17;8(1):76. doi: 10.1186/s40643-021-00428-9 (PMC10992110; doi:10.1186/s40643-021-00428-9)
Supplement: Supplementary file 1 — Additional file 1. 1) Materials, 2) Analysis and quantification of cellulolytic enzymes, 3) Analysis of enzyme activity, 4) Determination of isoflavonoids using high-performance liquid chromatography coupled UV–Vis detector, 5) Determination of miroestrol and deoxymiroestrol using indirect competitive enzyme-linked immunosorbent assay (icELISA), and 6) optimization of microwave-assisted extraction. Fig. S1. SDS-PAGE of the cellulolytic enzyme from Trichoderma reesei. Lane 1 shows protein molecular mass markers, and the other lane (lane 2) shows cellulolytic enzymes. Fig. S2. Chromatograms of authentic compounds (a), including PUE, DZ, GT, DZe, and GTe, and the compounds extracted using 80% ethanol (b), ChCl:PG without cellulolytic enzymes (c), and ChCl:PG with cellulolytic enzymes (d). Fig. S3. The calibration curves of miroestrol via icELISA. Fig. S4. The catalytic activity of cellulolytic enzymes at 40 mU/mL (b), 100 mU/mL (c), and 500 mU/mL (d) with PMIs (PUE, DZ, GT, DZe, and GTe), where control (a) was performed without the enzyme. The *indicates a significant difference compared to the concentration at the initial time (p < 0.05). Fig. S5. The extraction and biotransformation of PMIs using cellulolytic enzymes with different NADESs, including 20% ChCl:G (a), 40% ChCl:G (b), 20% ChCl:PG (c), and 40% ChCl:PG (d), in which the reactions were conducted in the temperature range of 30, 50, and 70 °C. The same and different letters indicate nonsignificant and significant differences in the extractive yield of each compound between the temperature treatments, respectively. Statistical significance was determined by one-way ANOVA, followed by LSD (p < 0.01). Table S1. The effect of microwave power on the yields of PMIs extracted by MAE. Table S2 The effect of irradiation time on the yields of PMIs extracted by MAE. Table S3. The experimental variable factors of the BBD for the extraction of PMIs from PM. Table S4. The rate of daidzein production using cellulolytic enzy [file 40643_2021_428_MOESM1_ESM.docx]

Additional file 1

**Biocompatible natural deep eutectic solvent-based extraction and cellulolytic enzyme-mediated transformation of *Pueraria mirifica* isoflavones: A sustainable approach for increasing health-bioactive constituents**

Fonthip Makkliang^1, †^, Boondaree Siriwarin^2, †^, Gorawit Yusakul^3, 4^*, Suppalak Phaisan^3^, Attapon Sakdamas^3^, Natthapon Chuphol^5^, Waraporn Putalun^6^, Seiichi Sakamoto^7^

*^1^School of Languages and General Education, Walailak University, Nakhon Si Thammarat, Thailand*

*^2^Faculty of Pharmaceutical Sciences, Huachiew Chalermprakiet University, Samut Prakan, Thailand*

*^3^School of Pharmacy, Walailak University, Nakhon Si Thammarat, Thailand*

*^4^Biomass and Oil Palm Center of Excellence, Walailak University, Nakhon Si Thammarat, Thailand*

*^5^Faculty of Pharmaceutical Sciences, Prince of Songkla University, Songkhla, Thailand*

*^6^Faculty of Pharmaceutical Sciences, Khon Kaen University, Khon Kaen, Thailand*

*^7^Graduate School of Pharmaceutical Sciences, Kyushu University, Higashi-ku, Fukuoka, Japan*

*Corresponding author: Gorawit Yusakul

School of Pharmacy, Walailak University, Nakhon Si Thammarat 80160, Thailand

Tel: +66(0)75-67-2839, Email address: [gorawit.yu@mail.wu.ac.th](mailto:gorawit.yu@mail.wu.ac.th), [gorawit.yu@wu.ac.th](mailto:gorawit.yu@wu.ac.th)

^†^These authors contribute equally.

1. **Materials**

*Pueraria candollei* var. *mirifica* (Airy Shaw & Suvat.) Niyomdham (PM) roots were provided by DOD Biotech Pub Co., Ltd. (Samut Songkhramm, Thailand). The PM roots of six-year-old cultivation were collected from Suphan Buri Province (Thailand). After collection from the field, the roots were sliced into thin pieces and dried under sunlight. Additional drying was performed using an oven (50°C) for 24 hours. The PM roots were ground and kept at 4°C until the experiment. The cellulolytic enzyme (*Trichoderma reesei*) was purchased from Xi'an Biof Bio-technology Co., Ltd. (Shaanxi, China). Puerarin (PUE, 99.2%), daidzein (DZe, 97.3%), and genistein (GTe, 99.3%) were purchased from LKT Laboratories, Inc. (MN, USA). Genistin (GT, 99%) was from Fujicco Co., Ltd. (Tokyo, Japan). Daidzin (DZ, 97.3%), 4-nitrophenyl β-D-glucopyranoside (*p*NPG), and *p*-nitrophenol (*p*NP) were obtained from Sigma-Aldrich (MO, USA). Miroestrol (MI) and deoxymiroestrol (DMI) were purified from PM roots (Yusakul et al. 2020).

1. **Analysis and quantification of cellulolytic enzymes**

SDS-PAGE analyzed the cellulolytic enzyme. The proteins were separated by 12.5% (w/v) SDS-PAGE under reducing conditions and then stained with Coomassie Brilliant Blue reagent (Loba Chemie Pvt. Ltd.). The molecular masses of the proteins in each fraction were estimated according to a standard protein marker (Enzmart Biotech, Thailand). The results are shown in Fig. S1. The cellulolytic enzymes are expected to be composed of β-D-glucosidase I (BGL I, ≈ 75 kDa), cellobiohydrolase I (CBH I, 59–68 kDa), and cellobiohydrolase II (CBH II, 50–58 kDa) (Kunamneni et al. 2014). Protein concentrations of cellulolytic enzymes were determined by the Bradford assay. The protein standard curve was prepared using bovine serum albumin (BSA) dissolved in phosphate-buffered saline (PBS). A set of dilutions ranging from 0 to 250 µg/ml BSA was used to generate a calibration curve. For each well of the microplate reaction, 20 µL of BSA solution or sample was mixed with 180 µL Bradford reagent in 96-well plates and incubated at room temperature for 20 min. The absorbance was measured at 595 nm using a microplate reader (Eon™, BioTek Instruments, Inc., VT, USA). The absorbances of the BSA reaction were plotted against the BSA concentration to establish the calibration curve.


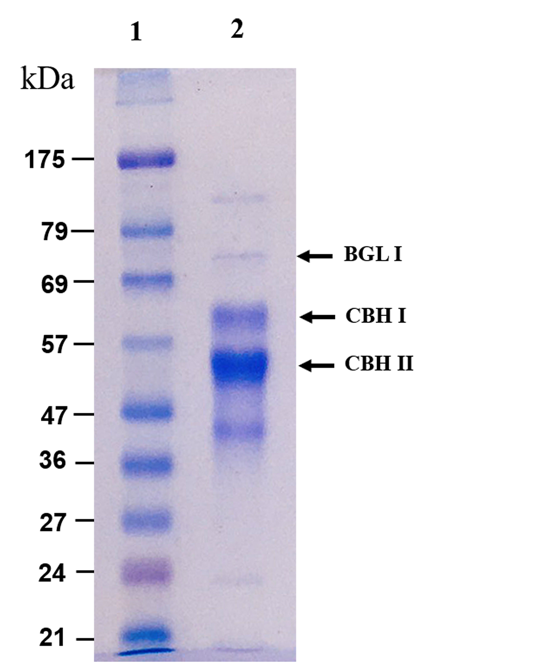


**Fig. S1** SDS-PAGE of the cellulolytic enzyme from *Trichoderma reesei*. Lane 1 shows protein molecular mass markers, and the other lane (lane 2) shows cellulolytic enzymes.

1. **Analysis of enzyme activity**

The cellulolytic enzyme was used to cleave the sugar moiety of DZ (DZe-7-O-β-D-glucoside) and GT (GTe-7-O-β-D-glucoside). Initially, the β-glucosidase activity of the cellulolytic enzyme was determined using *p*NPG as a chromogenic substrate (Matsuura, Sasaki and Murao 2014). The enzyme was dissolved in 50 mM sodium citrate-phosphate (pH 5) (SCP) buffer. The reaction was performed in a 96-microplate well; the mixture contained 50 µL of enzyme solution and 50 µL of *p*NPG. After incubation at 30°C for 10 min, the reaction was stopped with 100 µL of 50 mM sodium carbonate buffer pH 9.6. The absorbance was measured by using a microplate reader at 405 nm. The concentration of the product was calculated using the calibration curve of *p*-NP. The experiments were performed in triplicate. One unit (U) of the enzyme is the amount of enzyme that catalyzes the reaction of 1 µmol of *p*NPG per minute.

1. **Determination of isoflavonoids using high-performance liquid chromatography coupled UV-Vis detector**

In this study, the extraction processes yielded the isoflavonoids, including puerarin (PUE), daidzin (DZ), genistin (GT), daidzein (DZe), and genistein (GTe), which were determined using HPLC (Thermo Scientific Dionex Ultimate 3000, Thermo Scientific, MA, USA)). The HPLC method was modified from previous research(Yusakul et al. 2020). The mobile phases were gradient with a constant flow rate (1.0 mL/min) to separate the isoflavonoids using a reverse-phase analytical column (VertiSep™ USP C18 HPLC column, 4.6 mm × 250 mm, 5 µm particle size; Vertical Chromatography Co., Ltd., Nonthaburi, Thailand). The mobile phase consisted of 1.0% acetic acid (A) and 60% acetonitrile (B). The elution consisted of steps of 30% to 40% B (0-7 min) and 40% to 70% B (7-10 min), and then 70% B was maintained until 25 min. Solvent B was increased to 100% B to elute compounds from the matrix; after that, the column was re-equilibrated with 30% B for the next analysis. Eluted compounds were detected by an ultraviolet (UV) detector at 254 nm. Chromatograms of authentic compounds and samples are shown in Fig. S2.


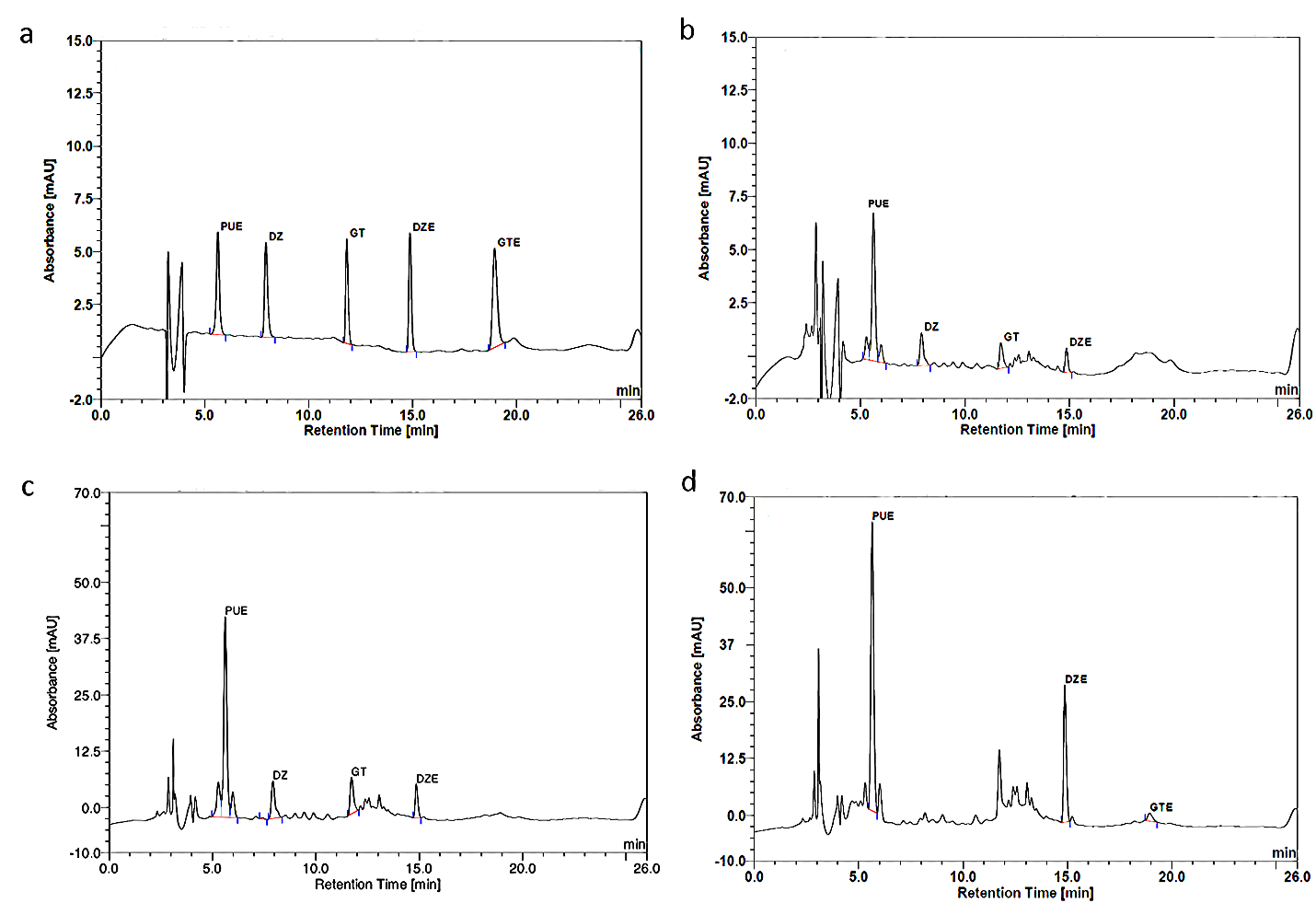


**Fig. S2** Chromatograms of authentic compounds (a), including PUE, DZ, GT, DZe, and GTe, and the compounds extracted using 80% ethanol (b), ChCl:PG without cellulolytic enzymes (c), and ChCl:PG with cellulolytic enzymes (d)

1. **Determination of miroestrol and deoxymiroestrol using indirect** **competitive enzyme-linked immunosorbent assay (icELISA)**

The icELISA procedure was the same as that in a previous report (Yusakul et al. 2018). The miroestrol-HSA conjugate (1 μg/mL, 100 L) was diluted in 50 mM sodium carbonate buffer pH 9.6 and incubated for one hour in a 96-well plate. The plate was then treated with 5% (w/v) skim milk in phosphate-buffered saline (PBS). In the next step, aliquots (50 µL) of miroestrol solution were added to each well. Then, the primary antibody (0.25 µg/mL, 50 µL) was allowed to react competitively between immobilized and free miroestrol in each well for one hour. The plate was then washed to eliminate any remaining unfixed antibodies. Secondary antibodies (0.5 μg/mL, 100 µL) were reacted with antibodies bound to immobilized miroestrol for one hour. After three more washes, a substrate solution [0.003% (v/v) H_2_O_2_ and 0.3 mg/mL 2,2′-azino-bis(3-ethylbenzothiazoline-6-sulfonic acid) diammonium salt (ABTS) in 100 mM sodium citrate buffer (pH 4.0)] was added to each well, and the mixture was incubated for 15 minutes. With a microplate reader, absorbance at 405 nm was measured. At 37°C, all reactions were carried out. Miroestrol at a concentration of 0.625 to 10.0 µg/mL was used to generate a calibration curve (Fig. S3). As mentioned in previous research (Yusakul et al. 2018), the monoclonal antibody (mAb) utilized recognizes both miroestrol and deoxymiroestrol to a similar degree. icELISA could be used to quantify the total amount of MI and DMI in the PM extracts. The binding specificity of the mAb was rechecked, and the cross-reactivities toward DMI, isomiroestrol, and 7-O methylisomiroestrol were 120, 6.80, and 0.57%, respectively. As reported previously, the binding specificity of the mAb was similar (Yusakul et al. 2018).


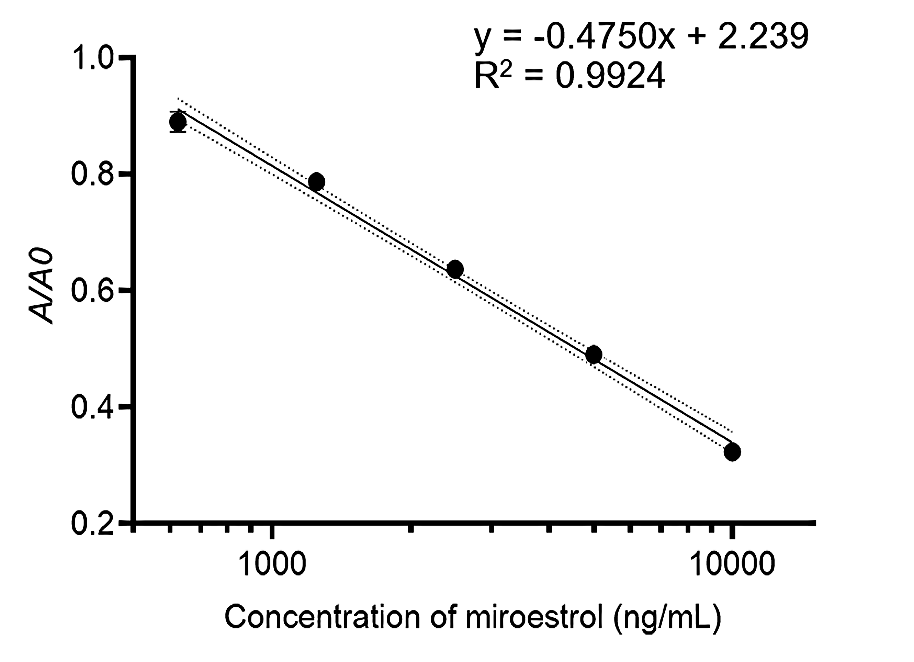


**Fig. S3** The calibration curves of miroestrol via icELISA

1. **Optimization of microwave-assisted extraction**

Before optimization of the extraction parameters, microwave-assisted extraction (MAE) was initially optimized. PM root powder (25 mg) was suspended in two milliliters of 5% (v/v) ChCl:PG (1:2) containing 200 mU/mL cellulolytic enzyme. The mixture was incubated at 70°C for one hour, and then the mixture was microwaved for 5 seconds three times; microwave powers of 100, 180, 300, 450, 600, and 850 were evaluated. The extractant solution was collected after centrifugation at 5000 ×g for 5 min. The concentrations of PMIs were determined using the described HPLC methods. The results indicated that 450 W microwave power produced the highest extracted DZe (Table S1). The extracted yield of PUE differs nonsignificantly between microwave powers. Thus, the mentioned power was used for further optimization.

**Table S1** The effect of microwave power on the yields of PMIs extracted by MAE

| Microwave power  (W) | The yields of extracted PMIs (µg/g PM DW) | | | | |
| --- | --- | --- | --- | --- | --- |
|  | PUE | DZ | GT | DZe | GTe |
| 100 | 0.983±0.008 ×10^3^ ^a^ | ND | ND | 381±3.05 ^a^ | 158±6.66 ^a^ |
| 180 | 0.938±0.013 ×10^3^ ^a^ | ND | ND | 385±2.47 ^a, b^ | 143±1.35 ^b^ |
| 300 | 0.970±0.005 ×10^3^ ^a^ | ND | ND | 401±1.52 ^c^ | 137±3.07 ^b, c^ |
| 450 | 0.960±0.006 ×10^3^ ^a^ | ND | ND | 469±2.59 ^d^ | 128±5.89 ^c^ |
| 600 | 0.953±0.005 ×10^3^ ^a^ | ND | ND | 381±4.93 ^a^ | 81.0±2.54 ^d^ |
| 850 | 0.937±0.007 ×10^3^ ^a^ | ND | ND | 393±4.71 ^b,c^ | 77.3±1.53 ^d^ |

The same and different letters indicate nonsignificant and significant differences in extractive yield of the compound, respectively, when the independent factor is the microwave power levels. Statistical significance was determined by one-way ANOVA, followed by LSD (*p* <0.01). ND, not detectable.

PM root powder (25 mg) was suspended in two milliliters of 5% (v/v) ChCl:PG (1:2) containing 200 mU/mL cellulolytic enzyme. The mixture was incubated at 70°C for one hour, and then the mixture was microwaved for 15 – 60 seconds three times at a microwave power of 450 W. The extractant solution was collected after centrifugation at 5000 ×g for 5 min. The concentrations of PMIs were determined using the described HPLC methods. The results indicated that 15 s irradiation produced the highest yields of extracted DZe. The longer decreased the yields of compounds.

**Table S2** The effect of irradiation time on the yields of PMIs extracted by MAE

| Irradiation time (s) | The yields of extracted PMIs (µg/g PM DW) | | | | |
| --- | --- | --- | --- | --- | --- |
|  | PUE | DZ | GT | DZe | GTe |
| 15 | 1.078±0.013×10^3 a^ | ND | ND | 468±3.35 ^a^ | 132±7.74 ^a^ |
| 30 | 1.039±0.005×10^3 b^ | ND | ND | 432±2.15 ^b^ | 128±2.96 ^a^ |
| 45 | 1.048±0.009×10^3 b^ | ND | ND | 426±12.6 ^b^ | 130±4.50 ^a^ |
| 60 | 1.063±0.003×10^3 a,b^ | ND | ND | 429±11.8 ^b^ | 134±2.47 ^a^ |

The same and different letters indicate nonsignificant and significant differences in extractive yield of the compound, respectively, when the independent factor is irradiation time. Statistical significance was determined by one-way ANOVA, followed by LSD (*p* <0.01). ND, not detectable.

1. **Supplementary tables**

**Table S3** The experimental variable factors of the BBD for the extraction of PMIs from PM

| **Factors** | **Symbol** | **Unit** | **Levels** | | |
| --- | --- | --- | --- | --- | --- |
|  |  |  | **Low (-1)** | **Medium (0)** | **High (1)** |
| **Independent variables** | | |  |  |  |
| - ChCl:PG concentration | *X_1_* | % | 5 | 20 | 35 |
| - Temperature | *X_2_* | °C | 30 | 60 | 90 |
| - Cellulolytic enzymes concentration | *X_3_* | mU/mL | 20 | 200 | 380 |
| **Dependent variables** | | |  |  |  |
| - Daidzein (DZe) yield | *Y_1_* | µg/g |  |  |  |
| - Genistein (GTe) yield | *Y_2_* | µg/g |  |  |  |

**Table S4** The rate of daidzein production using cellulolytic enzymes

| Enzyme concentration | Rate of daidzein production (µg/mL·min) | R^2^ of the linear regression |
| --- | --- | --- |
| 40 mU/mL | 0.3196 | 0.9968 |
| 100 mU/mL | 0.5617 | 0.9924 |
| 500 mU/mL | 1.405 | 0.9771 |

**Table S5** The effect of ChCl:PG concentration on PMI extraction and transformation efficiency

| The concentration of ChCl:PG, (%, v/v) | The yields of extracted PMIs (µg/g PM DW) | | | | |
| --- | --- | --- | --- | --- | --- |
|  | PUE | DZ | GT | DZe | GTe |
| 5 | 1.04±0.006 ×10^3 a^ | ND | 2.53±0.18 ^a^ | 414±0.92^a^ | 78.9±0.43 ^a^ |
| 10 | 1.05±0.010 ×10^3 a^ | ND | 2.06±0.53 ^a^ | 429±6.78^a^ | 77.6±0.85 ^a,c^ |
| 20 | 1.11±0.005 ×10^3 b^ | ND | 1.98±0.09 ^a^ | 459±5.72^b^ | 82.5±0.74 ^b^ |
| 30 | 1.13±0.014 ×10^3 b^ | 14.9±0.81 ^a^ | 2.11±0.02 ^a^ | 426±2.38^a^ | 81.4±0.24 ^b^ |
| 40 | 1.12±0.015 ×10^3 b^ | 57.8±1.36 ^b^ | 5.68±0.09 ^b^ | 381±1.86^c^ | 79.7±0.75 ^a^ |
| 50 | 1.12±0.016 ×10^3 b^ | 115±1.40 ^c^ | 15.3±1.76 ^c^ | 257±3.40^d^ | 75.8±0.62 ^c^ |

The PMIs were extracted from PM (100 mg) using various concentrations of ChCl:PG (1:2, 5 mL), where the other parameters were fixed at 300 mU/mL cellulolytic enzymes, one hour, and 50°C. The same and different letters indicate nonsignificant and significant differences in the extractive yield of each compound, respectively. Statistical significance was determined by one-way ANOVA, followed by LSD (*p* <0.01). ND, not detectable.

**Table S6** The effect of temperature on the PMI extraction and transformation efficiency

| Temperature  (°C) | The yield of extracted PMIs (µg/g PM DW) | | | | |
| --- | --- | --- | --- | --- | --- |
|  | PUE | DZ | GT | DZe | GTe |
| 40 | 1.02±0.009 ×10^3^ ^a^ | 19.1±0.39 ^a^ | 2.13±0.26 ^a^ | 391±9.63 ^a^ | 81.1±0.66 ^a^ |
| 50 | 1.05±0.010 ×10^3^ ^b,d^ | ND | 2.19±0.29 ^a^ | 465±4.32 ^b^ | 81.5±1.01 ^a^ |
| 60 | 1.11±0.001 ×10^3^ ^c^ | ND | 1.78±0.27 ^a^ | 483±1.24 ^b^ | 95.0±79.1 ^b^ |
| 70 | 1.04±0.011 ×10^3^ ^b^ | 9.33±0.38 ^b^ | 2.27±0.42 ^a^ | 430±7.28 ^c^ | 79.6±0.44 ^a^ |
| 80 | 1.07±0.008 ×10^3^ ^d^ | 129±2.04 ^c^ | 16.1±0.74 ^b^ | 265±1.67 ^d^ | 73.0±0.38 ^a^ |

The PMIs were extracted from PM (100 mg) using various temperatures, where the other parameters were fixed at ChCl:PG (1:2, 20% (v/v), 5 mL), 300 mU/mL cellulolytic enzymes, and one hour. The same and different letters indicate nonsignificant and significant differences in the extractive yield of each compound, respectively. Statistical significance was determined by one-way ANOVA, followed by LSD (*p* <0.01). ND, not detectable.

**Table S7** The effect of cellulolytic enzyme concentration on PMI extraction and transformation efficiency

| Cellulolytic enzymes (mU/mL) | The yield of extracted PMIs (µg/g PM DW) | | | | |
| --- | --- | --- | --- | --- | --- |
|  | PUE | DZ | GT | DZe | GTe |
| 100 | 1.02±0.009 ×10^3^ ^a^ | 10.7±0.21 ^a^ | 4.73±0.44 ^a^ | 460±6.60 ^a^ | 83.3±1.65 ^a^ |
| 200 | 1.05±0.003 ×10^3^ ^b^ | ND | 3.42±0.35 ^b^ | 473±1.44 ^a,b^ | 99.4±0.52 ^b^ |
| 300 | 1.04±0.015 ×10^3^ ^a,b^ | ND | 2.30±0.15 ^c^ | 480±0.63 ^b^ | 99.6±0.61 ^b^ |
| 400 | 1.13±0.014 ×10^3^ ^c^ | ND | 2.38±0.64 ^b,c^ | 491±6.69 ^b,c^ | 104±0.31 ^c^ |
| 500 | 1.08±0.010 ×10^3^ ^d^ | ND | 2.69±0.54 ^b,c^ | 490±1.44 ^c^ | 104±0.31 ^d^ |
| 600 | 1.10±0.009 ×10^3^ ^c,d^ | ND | 2.97±0.05 ^b,c^ | 504±6.60 ^c^ | 101±1.65 ^b,c,d^ |

The PMIs were extracted from PM (100 mg) using various concentrations of cellulolytic enzymes, where the other parameters were fixed at ChCl:PG (1:2, 20% (v/v), 5 mL), 60°C and one hour of extraction. The same and different letters indicate nonsignificant and significant differences in the extractive yield of each compound, respectively. Statistical significance was determined by one-way ANOVA, followed by LSD (*p* <0.01). ND, not detectable.

**Table S8** The effect of time on the PMI extraction and transformation efficiency

| Time  (min) | The yield of extracted PMIs (µg/g PM DW) | | | | |
| --- | --- | --- | --- | --- | --- |
|  | PUE | DZ | GT | DZe | GTe |
| 0 | 0.990±0.008 ×10^3^ ^a^ | 140±1.83 ^a^ | 20.1±0.92 ^a^ | 190±2.52 ^a^ | 49.6±0.11 ^a^ |
| 30 | 1.07±0.014 ×10^3^ ^b^ | 3.83±0.46 ^b^ | 7.96±1.56 ^b^ | 271±5.14 ^b^ | 103±2.55 ^b^ |
| 60 | 1.12±0.010 ×10^3^ ^c^ | ND | 3.33±0.18 ^c^ | 474±2.83 ^c^ | 104±0.63 ^b^ |
| 90 | 1.11±0.010 ×10^3^ ^c^ | ND | 1.84±0.54 ^c,d^ | 488±9.46 ^c,d^ | 105±1.87 ^b^ |
| 120 | 1.12±0.010 ×10^3^ ^c^ | ND | 1.38±0.54 ^c,d^ | 503±5.68 ^d^ | 106±2.35 ^b^ |
| 150 | 1.20±0.012 ×10^3^ ^d^ | ND | 1.10±0.42 ^d^ | 546±9.49 ^e^ | 102±2.05 ^b^ |

The PMIs were extracted from PM (100 mg) using various durations, where the other parameters were fixed at ChCl:PG (1:2, 20% (v/v), 5 mL), 60°C and 200 mU/mL cellulolytic enzymes. The same and different letters indicate nonsignificant and significant differences in the extractive yield of each compound, respectively. Statistical significance was determined by one-way ANOVA, followed by LSD (*p* <0.01). ND, not detectable.

**Table S9** The experimental design matrix of BBD and their results of daidzein (*Y_1_*) and genistein (*Y_2_*)

| Run | Independent variables | | |  | Responses | |
| --- | --- | --- | --- | --- | --- | --- |
|  | *X_1_* | *X_2_* | *X_3_* |  | *Y_1_* | *Y_2_* |
| 1 | 20 | 60 | 200 |  | 480±4.18 | 93.2±0.96 |
| 2 | 5 | 60 | 380 |  | 432±2.45 | 90.4±0.17 |
| 3 | 35 | 60 | 20 |  | 372±9.91 | 89.9±0.39 |
| 4 | 20 | 60 | 200 |  | 490±11.3 | 91.5±1.70 |
| 5 | 35 | 30 | 200 |  | 260±4.58 | 86.1±0.42 |
| 6 | 20 | 60 | 200 |  | 476±2.15 | 92.8±0.33 |
| 7 | 35 | 60 | 380 |  | 476±12.2 | 92.9±3.10 |
| 8 | 5 | 30 | 200 |  | 412±11.8 | 91.7±0.21 |
| 9 | 20 | 30 | 20 |  | 250±4.65 | 86.6±0.19 |
| 10 | 5 | 90 | 200 |  | 257±6.80 | 83.5±0.11 |
| 11 | 20 | 90 | 20 |  | 230±3.12 | 83.3±0.18 |
| 12 | 5 | 60 | 20 |  | 450±13.4 | 91.4±0.28 |
| 13 | 20 | 60 | 200 |  | 511±7.86 | 91.8±0.92 |
| 14 | 20 | 30 | 380 |  | 356±0.68 | 90.2±0.28 |
| 15 | 35 | 90 | 200 |  | 251±6.11 | 83.8±0.48 |
| 16 | 20 | 90 | 380 |  | 285±7.70 | 84.7±0.12 |
| 17 | 20 | 60 | 200 |  | 520±7.43 | 91.7±1.79 |

1. **Supplementary figures**
   1. **Fig. S4**

**
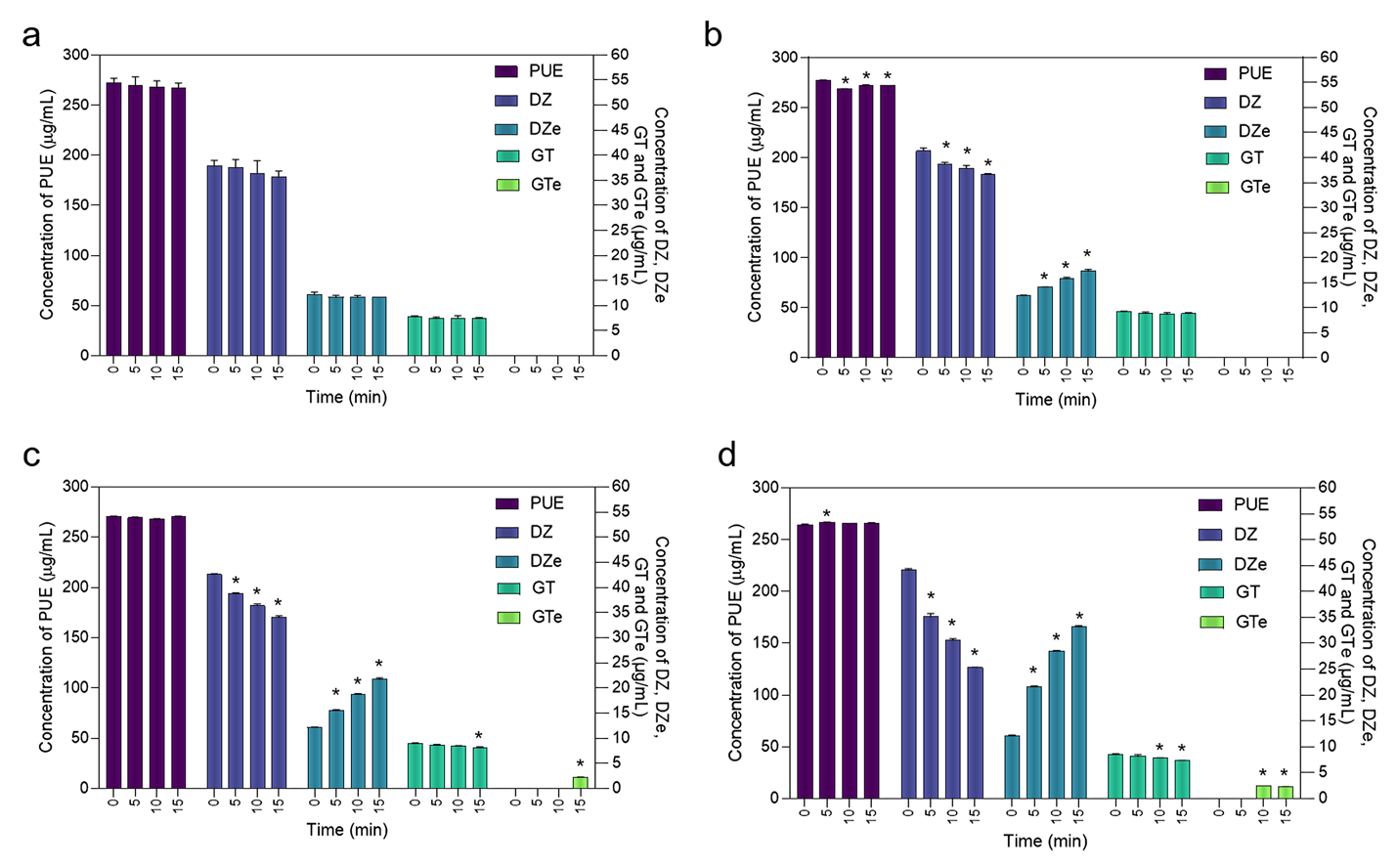
**

**Fig. S4** The catalytic activity of cellulolytic enzymes at 40 mU/mL (b), 100 mU/mL (c), and 500 mU/mL (d) with PMIs (PUE, DZ, GT, DZe, and GTe), where control (a) was performed without the enzyme. The * indicates a significant difference compared to the concentration at the initial time (*p* <0.05).

- 1. **Fig. S5**

**
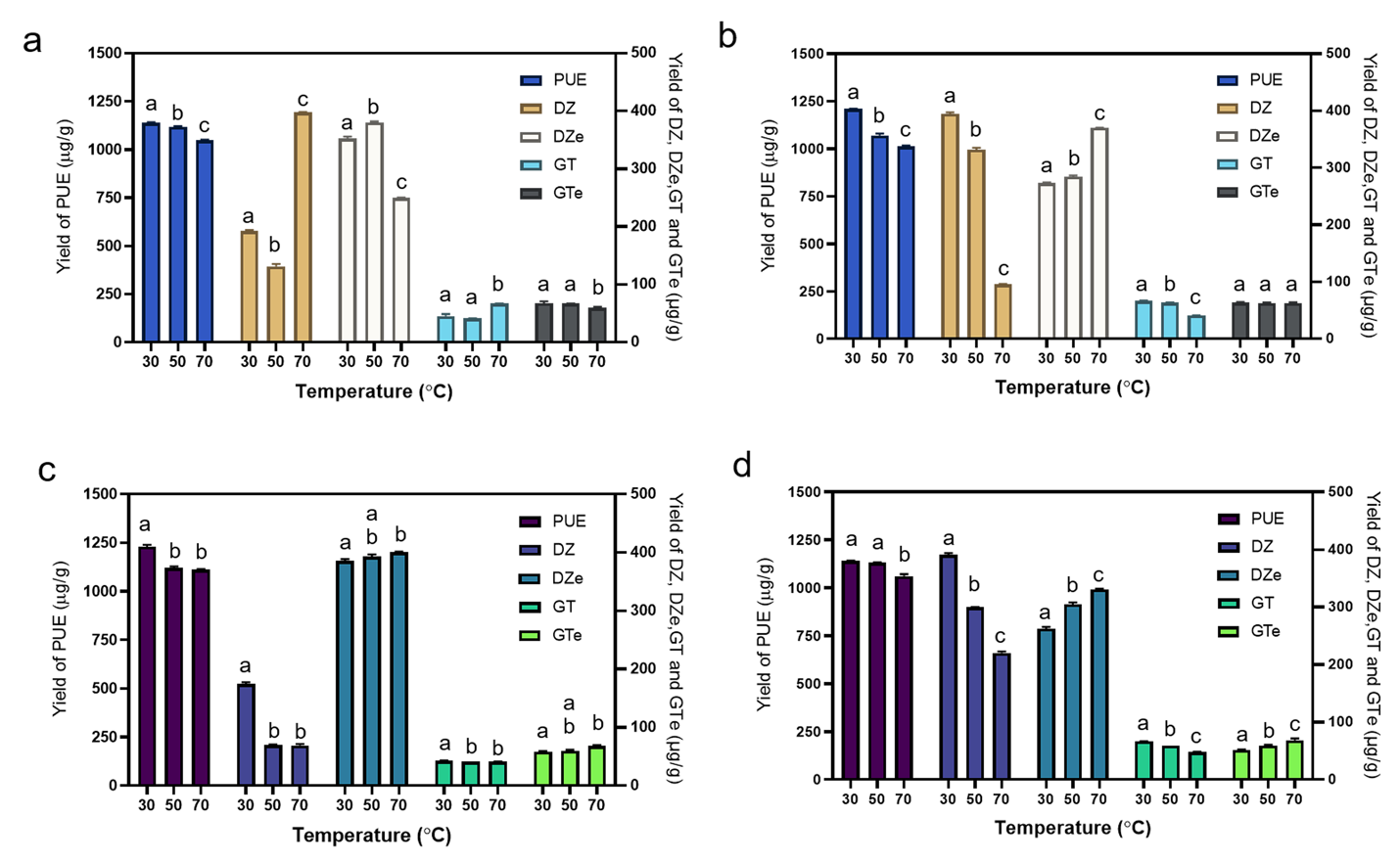
**

**Fig. S5** The extraction and biotransformation of PMIs using cellulolytic enzymes with different NADESs, including 20% ChCl:G (a), 40% ChCl:G (b), 20% ChCl:PG (c), and 40% ChCl:PG (d), in which the reactions were conducted in the temperature range of 30, 50, and 70°C. The same and different letters indicate nonsignificant and significant differences in the extractive yield of each compound between the temperature treatments, respectively. Statistical significance was determined by one-way ANOVA, followed by LSD (*p* <0.01)**.**

**References**

Kunamneni A, Plou FJ, Alcalde M, Ballesteros A (2014) *Trichoderma* Enzymes for Food Industries. In: Gupta VK, Schmoll M, Herrera-Estrella A, Upadhyay RS, Druzhinina I, Tuohy MG (Eds.) Biotechnology and Biology of *Trichoderma*, Elsevier, Amsterdam.

Matsuura M, Sasaki J, Murao S (2014) Studies on β-glucosidases from soybeans that hydrolyze daidzin and genistin: isolation and characterization of an isozyme. Biosci Biotechnol Biochem 59(9):1623-1627. <https://doi.org/10.1271/bbb.59.1623>.

Yusakul G, Juengsanguanpornsuk W, Sritularak B, Phaisan S, Juengwatanatrakul T, Putalun W (2020) (+)-7-O-Methylisomiroestrol, a new chromene phytoestrogen from the *Pueraria candollei* var. *mirifica* root. Nat Prod Res. <https://doi.org/10.1080/14786419.2020.1727473>.

Yusakul G, Kitisripanya T, Juengwatanatrakul T, Sakamoto S, Tanaka H, Putalun W (2018) Enzyme linked immunosorbent assay for total potent estrogenic miroestrol and deoxymiroestrol of *Pueraria candollei*, a Thai herb for menopause remedy. J Nat Med 72(3):641-650. <https://doi.org/10.1007/s11418-018-1194-x>.
